# Atrophic dermatofibrosarcoma protuberans: report of a case demonstrated by detecting COL1A1-PDGFB rearrangement

**DOI:** 10.1186/1746-1596-7-166

**Published:** 2012-11-30

**Authors:** Jianjun Qiao, Kayuri U Patel, Dolores López-Terrada, Hong Fang

**Affiliations:** 1 Department of Dermatology, The First Affiliated Hospital, College of Medicine, Zhejiang University, No. 79, Qingchun Road, Hangzhou, 310003, People’s Republic of China; 2 Department of Pathology, Texas Children’s Hospital and Baylor College of Medicine, Houston, TX, 77030, USA

**Keywords:** Dermatofibrosarcoma protuberans, COL1A1-PDGFB, CD34, Atrophic, Sarcoma

## Abstract

Dermatofibrosarcoma protuberans is a locally aggressive mesenchymal neoplasm. It usually presents as an indurated plaque that protrudes above the surface of the skin. Some patients have clinically persistent plaques that might be atrophic. The atrophic variant of dermatofibrosarcoma protuberans may be confused with some common skin diseases with atrophic appearance. We reported a 40-year-old woman who had a 10-year history of an atrophic dermatofibrosarcoma protuberans. Molecular analysis showed a fusion between *COL1A1* exon 31 to exon 2 of *PDGFB*. The lesion was totally excised, with negative margins of the resection demonstrated by CD34 immunostaining. To our knowledge, this is the second case of atrophic dermatofibrosarcoma protuberans confirmed by detection of COL1A1-PDGFB fusion gene. This appears to be the first report of a fusion between *COL1A1* exon 31 to exon 2 of *PDGFB* in atrophic dermatofibrosarcoma protuberans.

**Virtual slides:**

The virtual slides for this article can be found here: http://www.diagnosticpathology.diagnomx.eu/vs/1249657688795311

## Background

Dermatofibrosarcoma protuberans is a locally aggressive mesenchymal neoplasm [1]. The lesions of this tumor usually present as indurated plaques that protrude above the surface of the skin [1,2]. The atrophic variant is a rare form of the tumor [3-5]. Translocation of t(17;22)(q22;q13) is the cytogenetic features of dermatofibrosarcoma protuberans [6]. As a consequence of this chromosomal rearrangement, fusion of the collagen type I α 1 *(COL1A1)* gene on 17q, with the platelet-derived growth factor β-chain *(PDGFB)* gene on 22q, is detected [6]. The fusion gene has been detected in several unusual variants of dermatofibrosarcoma protuberans (pigmented variant, giant cell fibroblastoma, and granular variant) [1,6]. The fusion gene has been detected only in one case of atrophic variant of dermatofibrosarcoma protuberans [7]. Here we report a case of atrophic dermatofibrosarcoma protuberans confirmed by detection of the COL1A1-PDGFB fusion gene. The gene fusion between *COL1A1* exon 31 to exon 2 of *PDGFB* observed in our patient is firstly reported in atrophic dermatofibrosarcoma protuberans.

## Case presentation

**Figure 1:**
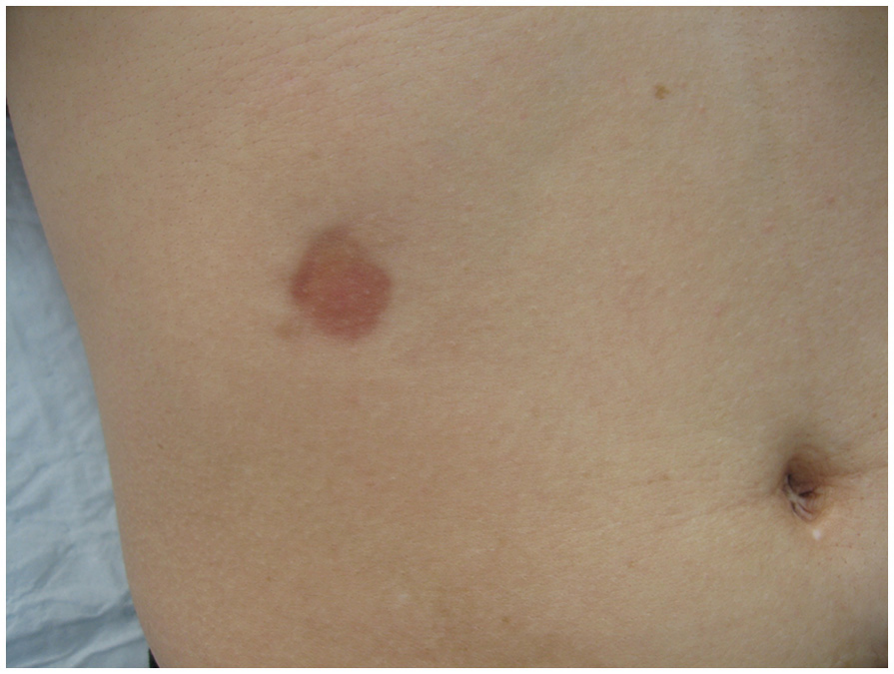
A smooth-surfaced, round, depressed plaque on the right pars lumbalis area.

**Figure 2:**
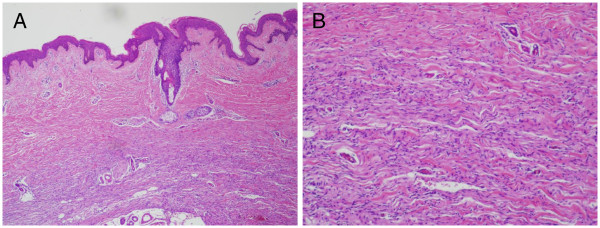
(A) Biopsy revealed a monomorphic spindle cell proliferation in the dermis and subcutaneous fat, with the spindle cells aligned horizontally to the epidermis section (hematoxylin-eosin, original magnification: ×100). (B) The nuclei of the spindle cells were thin, elongated and often wavy, with little pleomorphism (hematoxylin-eosin, original magnification: ×400).

**Figure 3:**
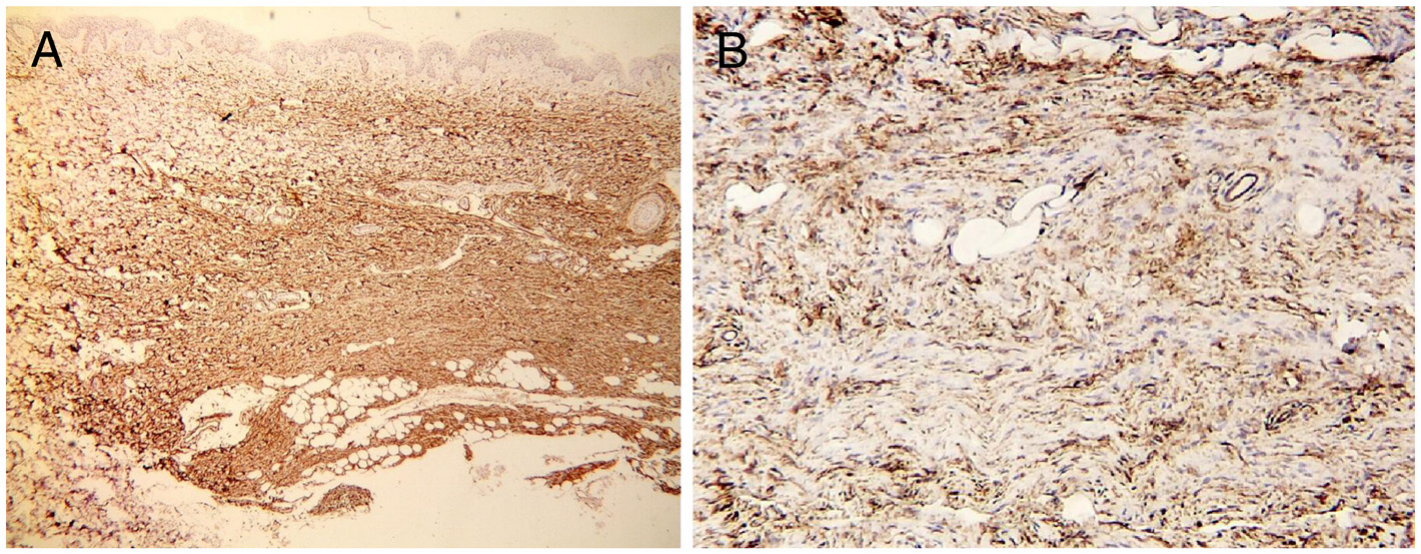
The spindle cells were immunohistochemically positive for CD34 (CD34 stain; original magnifications: A, ×40, B, × 400).

**Figure 4:**
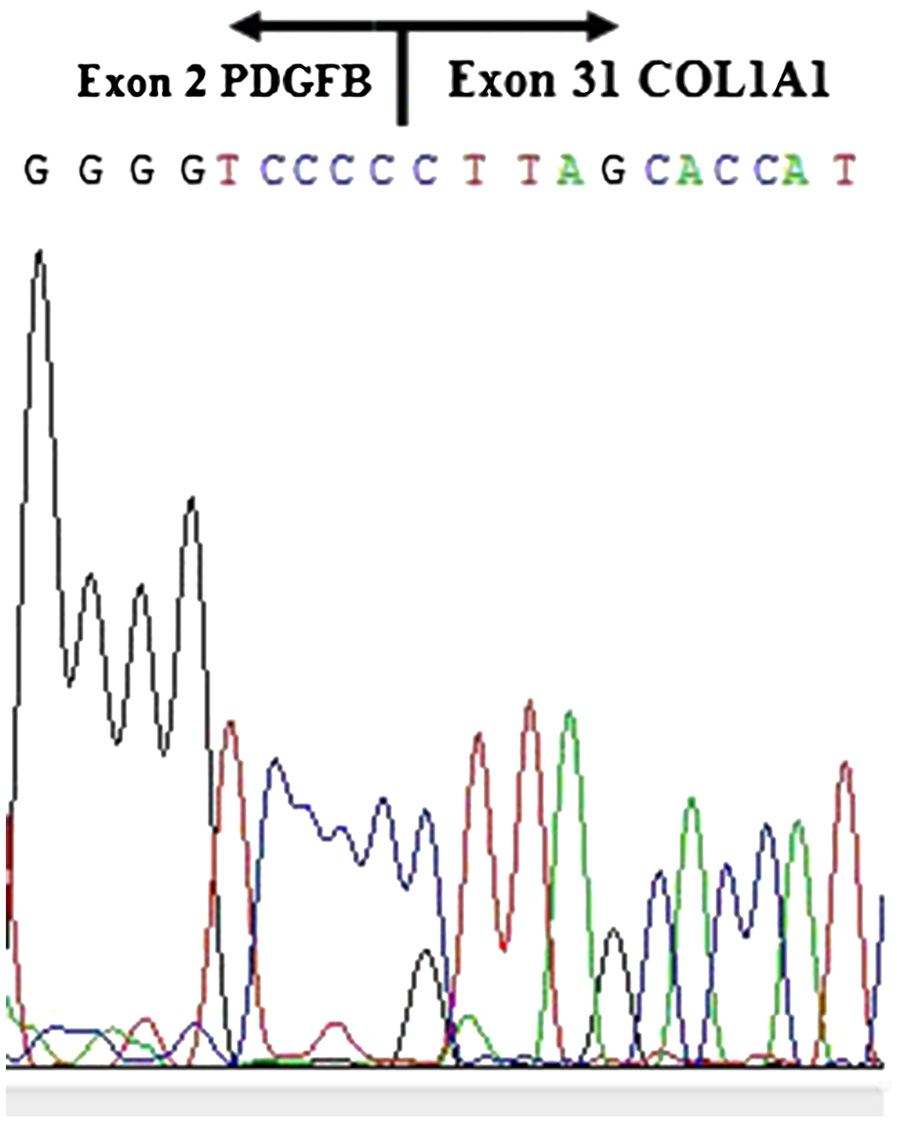
Detection of surgical specimen revealed a fusion of collagen 1 α1 gene (*COL1A1*) exon 31, to exon 2 of the platelet derived growth factor B gene (*PDGFB*).

In Apr 2010, a 40-year-old Chinese woman presented to our department of dermatology with a 10-year history of an asymptomatic, slowly enlarging rash located on the right pars lumbalis. She was otherwise healthy and had no systemic symptoms. The patient had no personal or family history of cancer. There was no history of trauma to the area. Physical examination showed a smooth-surfaced, round, depressed plaque on the right lumbalis area (Figure 1). On palpation the rash was atrophic with no induration. Clinical diagnoses of morphea and morpheaform basal cell carcinoma were suspected. The lesion was totally excised. Histopathology examination revealed extensive areas of tumor cell growth in the dermis and subcutaneous tissue. The tumor was infiltrating the surrounding tissue and had poorly defined margins. The tumor was composed of monomorphic spindle cells that aligned horizontally to the epidermis (Figure 2A). The nuclei were thin, elongated and often wavy, with little pleomorphism (Figure 2B). The tumor tissue contained no mucin and melanins. The spindle cells were immunohistochemically positive for CD34 (Figure 3) and vimentin, and negative for factor XIIIa, smooth muscle actin, and CD68. The surgical margins were negative demonstrated by CD34 immunostaining. RNA was extracted from the formalin-fixed, paraffin embedded surgical specimen for COL1A1/PDGFB chimeric transcripts analysis, by multiplex reverse transcription polymerase chain reaction (RT-PCR) assay. Sequencing of the multiplex RT-PCR amplification product revealed a fusion of exon 31 of *COL1A1*, to exon 2 of *PDGFB* (Figure 4). A diagnosis of atrophic dermatofibrosarcoma protuberans was established. The patient remains without evidence of local recurrence after 2 years of follow-up.

## Discussion

Dermatofibrosarcoma protuberans is a locally aggressive mesenchymal neoplasm with an intermediate grade and a high rate of local recurrence [1,6]. The incidence has been reported between 0.8–5.0 cases per 1 million persons per year. Men and women are equally affected. Dermatofibrosarcoma protuberans most commonly occurs in adults between the ages of 20 and 50, and are most commonly located on the trunk and the proximal extremities [1,2].

Dermatofibrosarcoma protuberans usually appears as an indurated, exophytic, asymptomatic, flesh-to-red/brown plaque that protrude above the surface of the skin [1]. These characteristic lesions are usually not difficult to diagnose clinically. However, the often slow clinical evolution of dermatofibrosarcoma protuberans is typified by an initial plaque stage and a later nodular stage [8]. Some patients have clinically persistent plaques that might be atrophic, as in our patient [5]. These atrophic plaques are believed to represent early lesions in the course of dermatofibrosarcoma protuberans [8,9], and have rarely been reported and characterized in detail. Given the typical ‘protuberant’ morphology, the diagnosis of atrophic lesions of dermatofibrosarcoma protuberans is frequently delayed [5,8].

The atrophic variant of this tumor is often clinically confused with common conditions characterized by atrophy or sclerosis, such as morphea, atrophic scar, morpheaform basal cell carcinoma, lipoatrophy, idiopathic atrophoderma, anetoderma, atrophic dermatofibroma, neurofibroma, resolving panniculitis, steroid atrophy, and medallion-like dermal dendrocyte hamartoma [3-5,8,10,11].

Histopathologically, dermatofibrosarcoma protuberans tumor cells are composed of densely packed, monomorphous spindle cells arranged in a storiform or whorled pattern, frequently infiltrating into subcutis and extending laterally into the surrounding dermis [1]. It may also involve the fascia, muscle and bone [6]. Cells of this tumor typically exhibit low mitotic activity [6]. There are some variants of dermatofibrosarcoma protuberans, including pigmented variant (Bednar tumor), congenital variant, giant cell fibroblastoma, atrophic variant, and fibrosarcomatous variant. The former four variant typically behave similarly to the classic type. However, the fibrosarcomatous variant is more aggressive, with a high propensity for metastasis [1,6].

Histopathologically, dermatofibrosarcoma protuberans should be differentiated from other cutaneous spindle cell tumors, including dermatofibroma, fibrosarcoma, malignant fibrohistocytoma, atypical fibroxanthoma, desmoplastic melanoma, keloid, Kaposi sarcoma, and solitary fibrous tumor [1,12,13]. Immunostains are helpful to differentiate dermatofibrosarcoma protuberans from other fibrohistiocytic neoplasms. Typically, cells of dermatofibrosarcoma protuberans are diffusely labeled with CD34 and are negative for factor XIIIa [1,5], distinguishing it from analogous neoplasms. The converse is true for most cases of dermatofibroma. Dermatofibrosarcoma protuberans is negative for CD68, allowing differentiation from atypical fibroxanthoma and malignant fibrohistocytoma, and is also typically negative for S100, helping distinguish it from desmoplastic melanoma [14].

Solitary fibrous tumor also consists of bland spindle cells but the tumor is well circumscribed. Similar to that in dermatofibrosarcoma protuberans, solitary fibrous tumor commonly labeled with CD34 and vimentin. However spindle cells in solitary fibrous tumor also express CD99 and bcl-2, which are negative for dermatofibrosarcoma protuberans [13].

Kaposi sarcoma is a vascular tumor with low-grade malignant potential. The tumor often composed of spindle cells, with slit-like vascular channels containing erythrocytes. The spindle cells not only express CD34 but also are immunoreactive for CD31 and human herpes virus 8 [12].

Medallion-like dermal dendrocyte hamartoma is a newly described and rare entity [15]. This tumor is benign. However, it shows morphologic, histological, and immunohistochemistrial overlap with atrophic dermatofibrosarcoma protuberans [15,16]. The lesions of medallion-like dermal dendrocyte hamartoma are round, solitary, well-demarcated, oval or triangular erythematous and atrophic plaques of several centimetres in diameter [15,16]. They are histologically characterized by a spindle cell proliferation in the dermis and in the subcutaneous fat. Furthermore, immunochemical staining for CD34 and factor XIIIa is usually positive in the hamartoma. Molecular testing is an important tool to differentiate these lesions from atrophic dermatofibrosarcoma protuberans, as there is not known rearrangements associated with hamartoma [15-17].

Nearly all dermatofibrosarcoma protuberans carry a t(17;22)(q22;q13) chromosomal translocation, which fuses the *COL1A1* gene on chromosome 17 to *PDGFB* on chromosome 22, and result in a chimeric COL1A1-PDGFB protein [18]. The translocation can be identified by fluorescence in situ hybridization, or RT-PCR, and confirmed by sequencing of the chimeric *COL1A1/PDGFB*. Llombart et al. reported the first case of atrophic dermatofibrosarcoma protuberans that was confirmed by COL1A1-PDGFB fusion gene detection [7]. To our knowledge, this is the second case of atrophic dermatofibrosarcoma protuberans whose diagnosis was confirmed by detection of the fusion gene. *COL1A1* encodes the α-chains of type 1 collagen, and *PDGFB* codes for the β-chain of platelet-derived growth factor. The latter is a potent mitogen that acts on a variety of cells. After gene fusion of *COL1A* and *PDGFB*, the inhibitory regulatory element of *PDGFB* is replaced by *COL1A1*, which allows for producing high levels of the chimeric COL1A1-PDGFB mRNA. PDGFB is cleaved from the COL1A1-PDFGB protein and the cleaved PDGFB turns to stimulate cells in an autocrine fashion, leading to malignant transformation [18]. Fusion of *COL1A1* exon 31 to exon 2 of *PDGFB*, as seen in our patient, was not reported previously in atrophic dermatofibrosarcoma protuberans. However, it has been postulated that the different types of fusion between *COL1A1* and *PDGFB* are not related with the clinical or histological features [7,19].

The standard treatment of the sarcoma is local excision with wide margins. Mohs surgery allows complete examination of the margins while sparing the maximum amount of healthy tissue [20-23]. Surgical treatment, however, is not always possible [20]. Imatinib mesylate, targeting platelet-derived growth factor receptor beta, has clinical potential in dermatofibrosarcoma protuberans. It has been demonstrated that imatinib mesylate inhibits the tyrosine kinase activity of PDGF-BB and causes apoptosis of the dermatofibrosarcoma protuberans cells [24,25]. Clinical trials demonstrated that imatinib has profound antitumor effects in advanced dermatofibrosarcoma protuberans harboring t(17;22) (q22;q13) [25-28].

## Conclusion

In conclusion, we report a case of atrophic dermatofibrosarcoma protuberans, which clinically masquerades as various atrophic cutaneous disorders. This variant is believed to represent an early stage of this lesion. This entity can be differentiated from other clinically similar lesions by histology, immunohistochemistry, and molecular genetics.

## Consent

Written informed consent was obtained from the patient for publication of this Case Report and any accompanying images. A copy of the written consent is available for review by the Editor-in-Chief of this journal.

## Abbreviations

COL1A1: Collagen type I α 1; PDGFB: Platelet-derived growth factor β-chain.

## Competing interests

The authors declare that they have no competing interests.

## Authors’ contributions

JQ looked after the patient. JQ and HF wrote the report. KUP and DL-T did the laboratory work. All authors read and approved the final manuscript. JQ is supported by the National Natural Science Foundation of China (30900056).
